# Profiling Communication Ability in Dementia: Validation of a new cognitive‐communication assessment tool

**DOI:** 10.1111/1460-6984.13153

**Published:** 2024-12-30

**Authors:** Suzanna Dooley, Tammy Hopper, Rachael Doyle, Orla Gilheaney, Margaret Walshe

**Affiliations:** ^1^ St. Columcille's Hospital Dublin Ireland; ^2^ Department of Clinical Speech and Language Studies Trinity College Dublin Dublin Ireland; ^3^ University of Alberta Edmonton Canada; ^4^ UCD Department of Geriatric Medicine, St. Vincent's University Hospital Dublin Ireland; ^5^ Clontarf Hospital Dublin Ireland

**Keywords:** cognitive communication, communication assessment, communication partner training, communication support strategies, dementia

## Abstract

**Background:**

Individuals with dementia have communication limitations resulting from cognitive impairments that define the syndrome. Whereas there are numerous cognitive assessments for individuals with dementia, there are far fewer communication assessments. The Profiling Communication Ability in Dementia (P‐CAD) was developed to address this gap.

**Aims:**

The purpose of this study was to examine the concurrent validity, longitudinal validity and inter‐ and intra‐rater reliability of the P‐CAD in a population of people with dementia and their communication partners.

**Method:**

The P‐CAD was administered to 122 people with dementia and their communication partners in Ireland (*n* = 100) and Canada (*n* = 22), over a 12‐month period. To establish concurrent validity of the P‐CAD, scores were compared to scores obtained from existing standardized instruments including the Functional Linguistic Communication Inventory (FLCI), the Mini‐Mental State Examination (MMSE‐2) and Global Deterioration Scale (GDS). Inter‐rater reliability and responsiveness (longitudinal validity) were analysed using data from a subgroup of participants.

**Outcomes & Results:**

Overall P‐CAD test scores were significantly correlated with FLCI (*n* = 122; *r* = 0.875; *p* < 0.001) and MMSE‐2 total scores (*n* = 122; *r* = 0.857; *p* < 0.001). Levels of communication support categories on the P‐CAD correlated with GDS rankings (*n* = 122; rho = −0.539; *p* < 0.001) and MMSE‐2 total scores (*n* = 122; rho = 0.680, *p* < 0.001). Inter‐rater reliability tested for 20 participants in the Irish sample revealed high levels of agreement between raters in scoring the GDS (*n* = 20; ICC = 0.969, *p* < 0.001), MMSE‐2 (*n* = 20; ICC = 0.997, *p* < 0.001), FLCI (*n* = 20; ICC = 0.999, *p* < 0.001) and P‐CAD (*n* = 20; ICC = 0.981, *p* < 0.001). To establish longitudinal validity to examine if the P‐CAD was responsive to changes in cognitive‐communication function over time, 12 participants in the Irish sample repeated all tests 3 months after the initial testing. No statistically significant differences in test scores were found for the 12 participants who completed follow‐up measures at this time point in any of the three scales. It was not possible to determine sufficient responsiveness as correlations between the change in P‐CAD scores over 3 months were insignificant for both the change in MMSE‐2 scores (rho = −0.130, *p* = 0.704) and the FLCI scores (rho = 0.221, *p* = 0.513).

**Conclusions & Implications:**

In this study, P‐CAD has demonstrated good concurrent validity and inter‐rater reliability in samples collected in two countries with English‐speaking participants. The P‐CAD is appropriate for use to evaluate communication abilities of people with dementia, including during conversational interactions with caregivers.

**WHAT THIS PAPER ADDS:**

**What is already known on the subject**

- Dementia alters the communication function of the person with dementia and impacts interactions with others. Speech and language therapists (SLTs) provide specific recommendations on communication function and support. However, they have limited access to comprehensive communication assessments to guide intervention.

**What this paper adds to the existing knowledge**

- This validation study has confirmed that Profiling Communication Ability in Dementia (P‐CAD) is a valid and reliable tool for SLTs to profile the communication abilities of people with dementia. It identifies the type and levels of communication support required as dementia progresses. The inclusion of communication partners in the study confirms their important role in providing conversation support to people with dementia.

**What are the potential or clinical implications of this work?**

- The P‐CAD is a clinical resource for dynamic communication assessment, which identifies key areas of retained ability to guide communication support and individualised intervention. It can be used with people at different stages and severity of dementia across a range of clinical settings. The P‐CAD summary, which is part of the P‐CAD, can be shared with family members and healthcare teams to enhance communication access for the person as dementia progresses.

## INTRODUCTION

Dementia is a neuro‐degenerative condition and a global health concern, affecting more than 55 million people worldwide (WHO, 2021). It is not a part of normal ageing, as healthy ageing is associated with only subtle declines in cognition (Harada et al., 2013; Salthouse, 2019) whereas cognition becomes significantly impaired in dementia to the extent that it interferes with daily life functioning (Elahi & Miller, 2017). In the most common forms of dementia, such as Alzheimer's disease, cognitive impairments in memory, attention, and executive functions cause disordered communication that begin as subtle deficits and become more pronounced and disabling over time as the dementia progresses. Communication is impaired in all forms of dementia; specific symptoms will vary depending on dementia type and personal factors unique to affected individuals (Hickey & Bourgeois, 2018).

Communication disorders have a significant impact on the relationship between the person with dementia and their family and friends. This can result in frustration, withdrawal from social interactions, depression, social isolation for the person with dementia (Wray, 2020) and increased caregiver burden (Chiao et al., 2015; Hickey & Bourgeois, 2018). Helping communication partners adapt to these changes in communication is essential for the person with dementia to maintain autonomy, and to connect meaningfully with others for as long as possible (Dooley et al., 2023). Thus, there is a recognised need to focus on communication assessment and intervention as part of the overall care of people living with dementia (Morris et al., 2018; Volkmer et al., 2018).

A valid reliable assessment to map communication impairment and its consequences is essential as part of the care pathway for people with dementia. A scoping review (Dooley & Walshe, 2019) identified gaps in available communication assessments in dementia and described the cognitive, linguistic and functional communication domains typically assessed by existing tools—highlighting a critical gap for people with dementia worldwide. Profiling Communication Ability in Dementia (P‐CAD) was developed to address the need for comprehensive communication assessments that could provide the following: (1) facilitate assessment of core communication competencies including auditory comprehension, verbal expression, reading and writing at different levels of function and difficulty, from single words to conversation; (2) include within the assessment process an opportunity to observe behaviours of a communication partner interacting with the person with dementia; (3) identify the level and type of communication support required to facilitate communication for the person with dementia; and (4) inform more specific communication interventions.

The P‐CAD assessment profiles the functional communication ability of individuals with dementia to provide a comprehensive profile of communication abilities, including interaction with a communication partner, to measure change over time and to guide communication intervention. The P‐CAD includes eight domains and associated subtests, including the following: attention, writing, auditory comprehension, verbal expression, conversation, reading and functional communication.

Development and refinement of the P‐CAD was guided by expert opinion and comprehensive feedback from user groups including people with dementia and their communication partners. This is described elsewhere (Dooley, 2020) and involves three key stages. The first stage involved taking a preliminary draft pilot version of the assessment and piloting it locally. Focus groups were recruited to establish the face, content, construct and ecological validity of this preliminary version of the assessment. Focus groups included people with dementia, their communication partners, health and social care professionals, physicians, nurses and speech and language therapists (SLTs). The second stage involved revising the preliminary assessment and conducting a pilot study involving SLTs working in dementia care who used the revised assessment. All participants answered questions related to the appearance, design, content and appropriateness and usefulness of the test. Final revisions were made in the third stage. These included the removal of culturally biased language and all dialect‐specific words/phrases. For example, the words ‘join the queue’ were replaced with ‘join the line’ and ‘cineplex’ replaced with ‘cinema’. The final version of the P‐CAD was then available for testing in the validation study that is described in this paper. An overview of the P‐CAD purpose and description subtests is available in Supporting information.

A unique feature of the P‐CAD is that the conversation section of the test has a provision for including a communication partner. In this section a conversation between the person with dementia and a communication partner is video recorded and two communication profiles are generated: one for the person with dementia and one for the communication partner. In addition, the P‐CAD yields a ‘communication support score’ that helps determine the level of communication assistance needed by the person with dementia. This rank‐ordered scale includes four levels of support: 0 (no support needed), 1 (minimum support), 2 (moderate support) and 3 (maximum support). Based on the participant's scores and overall communication profile, recommended communication strategies are provided.

The P‐CAD was devised to address a gap in communication assessments for people with dementia internationally. Although piloted originally with people living with dementia in Ireland, it was decided to extend data collection to a further site in North America to explore its potential applicability there. The purpose of this study was to examine the concurrent validity, longitudinal validity and inter and intra‐rater reliability of the P‐CAD in a population of people with dementia and their communication partners in English‐speaking populations in Ireland and Canada.

The following research questions were of interest: (1) What is the concurrent validity and reliability of data obtained from the P‐CAD? and (2) Is the P‐CAD responsive to changes in the cognitive‐communication ability of individuals with dementia over time?

## METHOD

Ethics approval was granted (Ref: TT78) from the local university in Ireland as well as from ethics committees at three hospital data collection sites. Ethics approval was also granted through the Health Research Ethics Board at the University of Alberta (Pro00071980) and Covenant Health Research Centre.

### Participants

The inclusion criteria for people with dementia included adults with a confirmed diagnosis of dementia by a physician, were proficient in English (determined by observation or caregiver report), were medically stable, and had hearing and visual skills sufficient to complete the testing in the designated testing environment based on study screening protocols). People with other neurological disorders (e.g., traumatic brain injury), a history of intellectual disability, severe dysarthria or a disorder marked by primary language deficits (e.g., primary progressive aphasia) were excluded from participating. Primary progressive aphasia affects communication in ways that are distinct from other common forms of dementia such as Alzheimer's disease and thus, the inclusion of individuals with this diagnosis was beyond the scope of this initial validation study. Communication partners were included if they were known to the individuals with dementia and had at least weekly contact with them. For the purposes of the study, when communication partners were unavailable to participate, a member of the research team acted in the role of communication partner.

### Recruitment

Recruitment was similar at both Irish and Canadian sites. The primary researcher at the research site presented information on the study in different healthcare settings and in Canada through the Alzheimer Society of Alberta and the Northwest Territories. After the presentations, researchers distributed consent forms to potential participants or provided the forms to staff who then mailed or emailed these to potential participants. In Ireland, gatekeepers (medical secretary, clinical nurse managers and SLTs) across research sites identified potential participants from caseloads. Accessible study information was distributed and once potential participants indicated that they were interested in participating in the study, the researcher obtained written informed consent directly from the participants and their legal guardians. This process was guided by the Assisted Decision‐Making (Capacity) Act (Oireachtas, 2015) in Ireland and Adult Guardianship and Trustee Act in Alberta (Alberta, 2009). Individuals with dementia had the opportunity to give consent through an adapted procedure. Specifically, consent to participate in the research study was documented by the individual's signature or mark if unable to write on the consent form or through verbal or non‐verbal assent (e.g., speaking aloud, pointing to the word ‘yes’ on a yes/no communication board). In all cases, written informed consent was obtained from the participant's legal decision‐maker on behalf of the person with dementia.

A sample size of 122 participants with dementia was calculated for a 5% level of significance and 80% power to determine if there is sufficient power to detect a meaningful difference in a given sample size. There was a conservative assumption of an equal percentage of responses across three categories of severity of cognitive decline (mild, moderate and severe).

A hundred individuals with dementia were recruited from community clinics and hospitals, as well as residential units affiliated with both acute and rehabilitation hospitals in Ireland. Additionally, 100 communication partners defined as care staff (family members, friends and significant others) were recruited. Twenty‐two people with dementia and 22 communication partners were recruited through the Alzheimer Society of Northern Alberta and the Northwest Territories, Canada as well as long‐term care settings within Alberta Health Services and Covenant Health.

Of the 122 participants with dementia in the study, the majority (62%) were women (*n* = 76). The average age of the people with dementia was 85 years (range 58–95 years), with years of education ranging from 8 to 18 years (median number of years: 12). Over half of the participants had a diagnosis of Alzheimer's disease, and severity of cognitive decline ranged from mild to severe/very severe cognitive decline (see Table 1).

**TABLE 1:** Participants’ gender, dementia subtypes and dementia severity rated on the Global Deterioration Scale (GDS).

	N (122)/%
Gender	
Men	46/38%
Women	76/62%
Dementia type	
Alzheimer	72/59%
Vascular	29/24%
Mixed	12/10%
Other	9/7%
Dementia severity (GDS rating)	
Level 4 (mild dementia)	61/50%
Level 5 (moderate dementia)	38/31%
Level 6 (moderately severe dementia)	17/14%
Level 7 (severe dementia)	6/5%

All participants (*n* = 122) had a communication partner present during testing. Communication partners who participated in the P‐CAD testing were typically family members, professional carers or friends who had at least weekly contact with the person with dementia, or an SLT researcher (if a communication partner was unavailable). The majority (75%) of communication partners were women (*n* = 92). In terms of the relationship to the person with dementia, 39 were spouses, 48 were professional carers, 21 were adult children, 12 were SLTs, one was a sibling and one was a friend of the participant.

### Data collection

After providing consent, individuals with dementia participated in a screening session to confirm eligibility for participation based on inclusion/exclusion criteria. Screening with the single word speech perception test (SWSPT) (Boothroyd, 1984) was done to ensure participants has adequate hearing to complete the assessments. A hearing amplifier was used with one participant and no participants were excluded based on hearing and vision status. The 2‐Question Instrument (Whooley, 2016) was also administered to screen for the presence of depression. Although the presence of depression was not an exclusion factor, data were collected using this test to allow for further exploration of the potential impact of depression on test performance.

**FIGURE 1:**
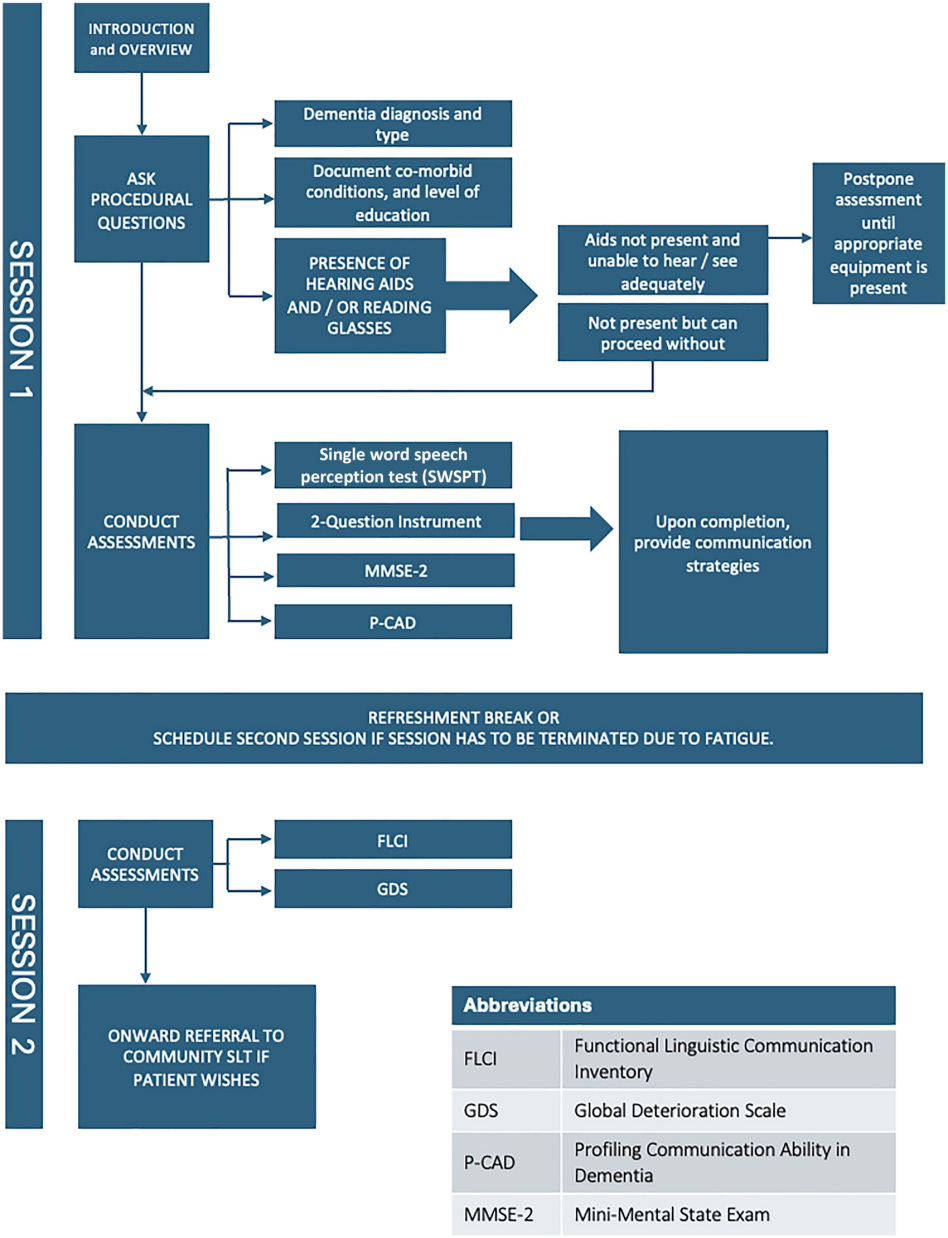
Data collection procedure. Abbreviation: SLT, speech and language therapist.

After completing the screening tests, participants with dementia completed the Mini‐Mental State Examination (MMSE‐2; Folstein et al., 2010) to assist in determining dementia severity and stage of cognitive decline on the Global Deterioration Scale (GDS; Reisberg et al., 1985). The P‐CAD and the reference test used for the assessment of concurrent validity, the Functional Linguistic Communication Inventory (FLCI) (Bayles & Tomoeda, 1994), were then administered (see Figure 1. Data collection procedure).

All data were collected by the researchers, SLTs familiar with the P‐CAD and experienced in administering standardised tests to adults who have dementia. Assessments were conducted in quiet, well‐lit rooms in the participant's place of residence or in the hospital outpatient department. The assessment process took approximately 90 min and was conducted in one sitting with a refreshment break. Where indicated, assessments were administered over two sessions on different days: for example, if the participants were fatigued or became unwell during data collection. Three participants in the Irish study were assessed on two different days due to illness (*n* = 1) and fatigue (*n* = 2).

### Assessment measures

#### P‐CAD

Administration of the P‐CAD takes approximately 30 min, and a total score of 24 is possible, with higher scores indicating better communication abilities.

#### FLCI

At the time of this study (2018–2019), the first edition of the FLCI (Bayles & Tomoeda, 1994) was used and the second edition (2022) had not yet been published. The FLCI is a long‐standing evaluation of the communication skills of individuals with moderate to more severe dementia. The FLCI has eight subtests and yields a total score of 84, with higher scores indicating better communication ability. It can be administered in 30 min. The established nature of the FLCI, as well as its similarity in purpose to the P‐CAD, made it an appropriate reference measure for the current study. Although similar in purpose, the FLCI was not designed for individuals with mild dementia, nor does it include extended cognitive domains assessed in the P‐CAD.

#### MMSE‐2

The MMSE‐2 (Folstein et al., 2010) has well‐established psychometric characteristics and includes items related to multiple cognitive constructs, including attention, orientation, working memory, episodic memory, executive functions, visuospatial abilities and language. The MMSE‐2 takes 5–8 min to complete, and scores may range from 0–30, with higher scores indicating better cognitive function.

#### GDS

The GDS (Reisberg et al., 1985) is an observational‐based scale that defines seven stages of cognitive decline associated with dementia. Rankings range from 1 (no cognitive decline) to 7 (very severe cognitive decline). The GDS was completed by the researchers based on the results of the MMSE‐2, observation of the person with dementia and interview with carers and family members.

### Data analysis

#### Concurrent validity

To confirm the concurrent validity of the P‐CAD, scores from 122 participants from Canada and Ireland were compared to the scores obtained from FLCI and the MMSE‐2 using Pearson Correlation Coefficients. Spearman's Rho was used to determine the relationship between the P‐CAD Level of Communication Support score and dementia severity as measured by the MMSE‐2 and the GDS.

#### Inter‐rater reliability

To test the degree to which different examiners would independently evaluate an individual's performance in the same way using the P‐CAD and other test measures, two SLT raters independently completed the P‐CAD, FLCI and MMSE‐2 on a sample of participants in the Irish sample. The SLT researchers took alternate roles of ‘tester’ and ‘observer’ for each administration session while sitting in the room with the participants. Both were blinded to the other person's rating on all the assessments. Both sets of test results were independently coded and compared for agreements using inter‐class correlation (ICC).

#### Responsiveness

Twelve people with dementia in the Irish sample were randomly selected using an online random number generator and invited by the primary researcher to be retested at a second time point. The time point selected was 3 months from the initial assessments. Only the P‐CAD, FLCI and MMSE‐2 were administered at this second time point by the same researcher. Differences in test scores over time were analysed using an extension of the Wilcoxon signed‐rank test (the Friedman Test).

## RESULTS

### Concurrent validity of P‐CAD

**FIGURE 2:**
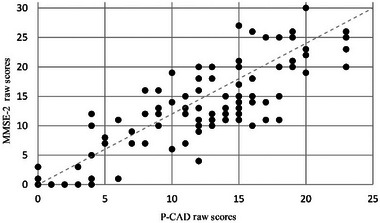
Profiling Communication Ability in Dementia (P‐CAD) correlation with Mini‐Mental State Examination (MMSE‐2).

**FIGURE 3:**
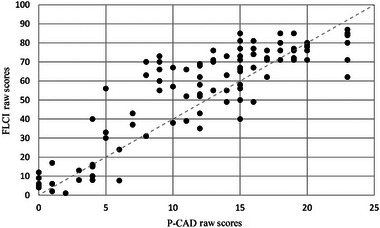
Profiling Communication Ability in Dementia (P‐CAD) correlation with Functional Linguistic Communication Inventory (FLCI).

The maximum score attainable on the MMSE‐2 is 30 and on P‐CAD is 24. Higher scores on the MMSE‐2 and P‐CAD are closer to the norm. A Spearman's rank‐order correlation was used to determine the relationship between P‐CAD, the MMSE‐2 and FLCI raw scores, as the scores were not normally distributed. Scores in communication ability on the P‐CAD correlated with cognitive functioning as measured by the MMSE‐2 (*n* = 122; *r* = 0.857; *p* < 0.00) (see Figure 2). Better communication function is also indicated by higher scores on the FLCI. There was a strong, positive correlation in communication ability scores between FLCI (*n* = 122; *r* = 0.875; *p* < 0.001) and P‐CAD (see Figure 3).

Level of Communication Support rankings on the P‐CAD significantly correlated with GDS rankings (*n* = 122; rho = −0.539; *p* < 0.001), suggesting that as dementia severity worsened, more communication support was needed. Similarly, participants with lower MMSE‐2 scores needed higher levels of communication support (*n* = 122; rho = 0.680, *p* < 0.001). For example, with a GDS ranking of 6–7, the participant needed maximum communication support (Level 3 Support) and with a GDS level of 4–5 the participant needed minimum communication support (Level 1 Support).

### Inter‐rater reliability of P‐CAD

Inter‐rater reliability was calculated for raters obtaining data from 20 participants. This revealed high levels of agreement in scoring the GDS (*n* = 20; ICC = 0.969, *p* < 0.001), MMSE‐2 (*n* = 20; ICC = 0.997, *p* < 0.001), FLCI (ICC = 0.999, *p* < 0.001) and P‐CAD (*n* = 20; ICC = 0.981, *p* < 0.001).

### Responsiveness

Changes in the cognitive‐communication ability of individuals with dementia over time (i.e., 3 months) were examined using the P‐CAD, the FLCI and MMSE‐2. There were no statistically significant changes in any of the three test scores over the 3‐month testing period for the 12 participants: MMSE‐2 scores (*n* = 12; rho = −0.130, *p* = 0.704) and the FLCI scores (*n* = 12; rho = 0.221, *p* = 0.513) were consistent with changes in scores on the P‐CAD.

## DISCUSSION

Concurrent validity and inter‐rater reliability were established for the P‐CAD in the current study. P‐CAD offers an alternative assessment for profiling the communication abilities of people with dementia (Dooley & Walshe, 2019). The validation of this assessment has clinical implications for guiding intervention across the stages of dementia when communication abilities are changing. Further analysis of P‐CAD communication support levels indicated a pattern of increased level of communication support as dementia severity worsens, as one would expect. P‐CAD support levels are categorised as minimum, moderate and maximum support. Following the validation study we know that these levels move in parallel with the stages of cognitive decline. This alignment of cognitive decline and the level of communication support required has the potential to inform care pathways and service delivery. The use of P‐CAD in the identification of individualised communication support strategies will guide SLTs in appropriate interventions with the progression of dementia. P‐CAD communication support strategies were used in a conversation coaching intervention for people with dementia (Dooley et al., 2023) which demonstrated a positive impact on communication and the well‐being of the person with dementia. During the P‐CAD assessment, people with dementia were directly involved in identifying communication support strategies and adapting their own interactions for better conversations. Identifying communication abilities is a primary assessment focus across P‐CAD communication domains. For example, the evaluation of expression includes review of non‐verbal communication, social engagement, language (verbal and written), participation, as well as the functional ability in phone use, emails and group conversations. This wide scope helps identify retained skills to maximise and enhance communication independence.

Data collection procedures should be flexible to ensure the comfort and engagement of participants with dementia in research. As mentioned earlier, in this study, the total assessment session was approximately 1.5 h with a 15‐min refreshment break. All participants were given this break time to reduce the impact of fatigue on testing. Ongoing verbal consent to proceed with assessments was obtained throughout. Three participants completed the assessments over 2 different days due to illness or fatigue. This is an important consideration when working with participants with dementia and it will be addressed in the design of any further validation studies. The involvement of the communication partner directly in the assessment is unique to P‐CAD and the review of conversation abilities undertaken can inform communication partner training interventions (Conway & Chenery, 2016; Volkmer et al., 2023) used by SLTs.

Change in cognitive‐communication ability in a small sample of participants, as measured by the P‐CAD and the reference tests, was not evident over 3 months. This study was unable to determine the responsiveness of P‐CAD at 3 months and research timelines did not allow for retest again at 6 months. However, this study has shown that P‐CAD yields reliable data across different testers and was appropriate for use in a Canadian context, with minor modifications to vocabulary items in the refinement phase.

## CONCLUSIONS

P‐CAD was developed and refined based on consultation with people who are living with dementia, their family members and healthcare professionals. This ensures that the assessment is relevant to the individuals for whom it is designed and provides data that can inform care pathways to better communication. Assessment feedback guides the communication partner in using individualised support strategies and promotes an understanding of communication strengths. This then facilitates the person with dementia to utilise their communication abilities and engage new strategies to enhance communication independence and social well‐being. The inclusion of caregivers in the assessment, with evaluation of their communication support skills while interacting with the person with dementia, is a unique aspect of the P‐CAD. If the person with dementia does not have a communication partner available during the assessment, the SLT or proxy may act as the partner and still gather important information about the conversational ability and communication function of the person with dementia.

With a limited number of functional communication assessments available for people with dementia, this study provides some evidence for the validity and reliability of the P‐CAD as a standardised test (Dooley et al., 2022) appropriate for English speakers with mild to severe cognitive decline as a result of dementia.

## LIMITATIONS AND FUTURE RESEARCH DIRECTIONS

The study as it stands has some limitations. While we could investigate in part the P‐CAD's sensitivity to change over time, the time period was likely too short for significant declines in cognition to occur as a result of the dementia process, in the absence of a medical event. Retesting cognitive‐communication abilities with the P‐CAD for a longer time period may have been a more suitable timeframe to profile cognitive‐communication changes related to dementia progression, but this was not logistically possible. Furthermore, even if there was a change in cognitive function over 3 months, the sample size was too small to yield adequate statistical power to detect such a change.

Another limitation of the study is that most of the participants with dementia were white, English‐speaking individuals. Although the sample was collected in two countries, the samples lacked the linguistic and racial diversity necessary to support the validation of P‐CAD in other cultural contexts. Future research should include validation of the P‐CAD in different languages and cultural contexts. As several cognitive tests are available in multiple languages, it may be appropriate to use these as reference tests and modify the P‐CAD as necessary based on the results.

Additionally, the samples comprised people with mostly Alzheimer's disease and excluded those with primary progressive aphasia and prominent language impairments. Future studies with larger samples of people with various dementia etiologies would be beneficial to support the use of the P‐CAD across dementia subtypes.

## CONFLICT OF INTEREST STATEMENT

The authors declare no conflicts of interest.

## Supporting information



Supporting Material

## Data Availability

The data that support the findings of this study are available on request from the corresponding author. The datasets generated and/or analysed in the current study are not publicly available due to privacy or ethical restrictions. Data collection was in line with the Data Protection Act (1998) which ensured that all information would be collected, stored and analysed in accordance with the Act, therefore, this data cannot be disclosed to anyone outside of the research team.
