# Supplementary material for: Profiling Communication Ability in Dementia: Validation of a new cognitive‐communication assessment tool
Source: Int J Lang Commun Disord. 2024 Dec 30;60(1):e13153. doi: 10.1111/1460-6984.13153 (PMC11684355; doi:10.1111/1460-6984.13153)
Supplement: Supplementary file 1 — Supporting Material [file JLCD-60-0-s001.docx]

**Supporting Information**

**Profiling Communication Ability in Dementia Overview**

**What is P-CAD?**

P-CAD is a functional cognitive communication test designed for individuals with dementia. It includes tasks that allow the clinician to evaluate core communication competencies of auditory comprehension, verbal expression, reading and writing. Specifically, P-CAD was developed to help speech and language therapists to do the following:

(1) evaluate the communication abilities of individuals with dementia,

(2) determine the effect of specific strategies to improve communication,

(3) develop a profile of communication strengths and weaknesses, and

(4) track changes in communication abilities over time and as a result of intervention.

It is intended that P-CAD will inform care pathways on communication, be of benefit to the interdisciplinary team in supporting decision-making and therefore ultimately have a positive impact on the well-being of the person with dementia and their families.

P-CAD can be used with people with different types of dementia, including Alzheimer disease, vascular dementia and mixed dementia, and across mild, moderate and severe stages of dementia. P-CAD focuses on identification of retained communication abilities of the person with dementia, to improve everyday conversations and quality of life, in all communication environments. Administration involves the person with dementia and their family member or primary carer, as communication partner (CP)

P-CAD evaluates the individual’s functional communication ability by screening eight cognitive communication domains.

| Section 1: Attention ability | Section 5: Writing ability |
| --- | --- |
| Section 2: Auditory comprehension ability | **Section 6: Conversation ability** |
| Section 3: Verbal expression ability | **Section 7: Communication support strategies ability** |
| Section 4: Reading ability | **Section 8:**  **Functional communication ability** |

**Section 1: Attention Ability**

Observe & rate the person’s attention during the assessment and while having a conversation

**Section 2: Auditory Comprehension Ability**

This section reviews auditory comprehension from word level to story comprehension level.

**Section 3: Verbal Expression Ability**

Evaluates verbal expression in naming, picture description and social exchanges.

**Section 4: Reading Comprehension Ability**

This section reviews the person with dementia’s reading comprehension from word level to long paragraph level. In the final subsection the person with dementia is asked to read a newspaper article. It is important for the clinician to reiterate to the person with dementia that this is not a memory task and that they can check back to the newspaper article to identify the answers.

**Section 5: Writing Ability**

A specific Writing Ability Form is provided for this section in the assessment booklet which records functional writing. The skills evaluated range from name signing, sentence generation, functional writing tasks, a shopping list and a greeting card.

**Section 6: Conversation Ability**

The therapist completes both Profile 1 & 2 with the person with dementia and their communication partner. This Conversation Ability Profile (CAP) can be used as a quick stand-alone assessment for conversation coaching. With permission, a short video recording is made and used as a basis for shared review of interactional styles, abilities and strategies to improve flow or troublesome patterns. The analysis of this short conversation includes the following areas; engagement, participation, resolving, sharing recognising potential and adjustments.

**Section 7: Communication Support Strategies**

This section evaluates the use of compensatory strategies by the person with dementia and their CP. Throughout the assessment session the clinician reviews how conversation is being supported with compensatory strategies and any patterns of communication breakdown. Communication support strategies are analysed under the headings of communication awareness, frequency and effectiveness of strategy use.

**Section 8: Functional Communication Ability**

This section is scored based on the clinician’s assessment of the person with dementia’s functional communication ability**.** The range and type of communication situations that the person engages in is evaluated. For example, how does the person with dementia manage in one-to-one and group conversations? What are their greatest communication strengths? Do they communicate mainly verbally or non-verbally? These questions facilitate a discussion about everyday functional communication between the person with dementia and their CP. The clinician records the responses in the table and rates functional communication ability.
